# Family Members’ Psychosocial Support in Palliative Inpatient Care: A Cross-Sectional Study

**DOI:** 10.1177/10748407251357063

**Published:** 2025-09-01

**Authors:** Anu Soikkeli-Jalonen, Kaisa Mishina, Johanna Saarinen-Nassar, Pauliina Kesonen, Heli Virtanen, Max Karukivi, Elina Haavisto

**Affiliations:** 1 Tampere University, Finland; 2 University of Turku, Finland; 3 Turku University Hospital, Finland; 4 Tampere University Hospital, Finland

**Keywords:** assessment, caregiver, end-of-life, family support, hospital, instrument, measurement, needs, palliative care, questionnaire, relatives

## Abstract

To evaluate the family members’ psychosocial support during specialist palliative inpatient care. Cross-sectional study. The “Family Involvement Scale–Psychosocial Support in Palliative Inpatient Care” (FIS-PS-InPal) questionnaire was used to collect data from family members from specialized palliative care wards across Finland. Data were analyzed using descriptive statistics. In total, 171 family members from 16 wards across Finland participated in the study. Family members evaluated the support they received as close to ideal, with 19.9% of participants reporting that the support was less than desired. They rated psycho-emotional support and support practices as the strongest and informational support as the weakest. The psychosocial support that family members receive in specialist palliative care is generally adequate. However, efforts should be made to improve how information is communicated. The study underscores the importance of evaluating and addressing the unique support implementation for families in palliative care.

## Contributions to the Global Clinical Community

- This study emphasizes the need for personalized support to address family members’ needs. The findings can help health care professionals improve communication and develop tailored support programs, enhancing family well-being.
- Health care professionals should proactively address the emotional and informational needs of family members to enhance psychosocial support. The study underscores the critical role of clear, factual, and truthful communication with family members when the patient is cared for in hospital.
- The insights from this study can guide policymakers and administrators in improving palliative care support systems, leading to more effective guidelines and policies that prioritize family involvement.

## Introduction

Globally, an estimated 56.8 million people require palliative care annually, the majority (67.1%) being adults aged above 50 (Connor, 2020). The illness and circumstances of a palliative patient impact the entire family. Hence, patients are often accompanied by numerous family members, leading to a significant number of individuals requiring palliative care services, both during the patient’s illness and following their passing (Connor, 2020).

Palliative care aims to prevent and alleviate the suffering experienced by both patients and their family members by addressing issues beyond physical symptoms (World Health Organization [WHO], 2022). Hence, the needs of family members should be acknowledged and addressed alongside those of the patient (Soikkeli-Jalonen et al., 2022, 2023). Consequently, comprehensive psychosocial support is vital in palliative care to aid family members in coping with and adjusting to the challenges presented by the situation (Soikkeli-Jalonen et al., 2022; Wang et al., 2018). According to the National Cancer Institute of the United States, psychosocial support comprises the mental, emotional, social, and spiritual needs of patients and their families (National Cancer Institute, 2024). Understanding family support, identifying family needs, and exploring opportunities to provide support are crucial considerations for the well-being of patients and families facing the challenges of a life-threatening illness (Wang et al., 2018).

As hospitals are the place of care and death for some individuals, enhancing people’s experiences of palliative care must be a part of the vision and mission of hospital organizations (Walker et al., 2023). However, current research regarding family members in palliative care predominantly focuses on home-based care (Walker et al., 2023), which limits its applicability to hospital settings (Ullrich et al., 2021). Moreover, studies regarding psychosocial support for families are limited (Soikkeli-Jalonen et al., 2021; Yıldız et al., 2024), underscoring the need for additional research to measure and evaluate how analyzing family needs can inform clinical practice to support families in palliative inpatient care more efficiently.

This study focuses on evaluating implemented psychosocial support of adult family members of adult patients in palliative inpatient settings in relation to their desired support. It aims to identify strengths and weaknesses in the current support system and propose improvements to enhance the support experienced by family members.

### Background

Family members dedicate substantial time and effort to care for palliative care patients (Areia et al., 2019). All severe illnesses place a burden on family members, a challenge that becomes more pronounced in the context of palliative care and hospital settings (Walker et al., 2023). The palliative care hospital environment distinguishes itself from other care settings, and family members have several needs, such as coping with the palliative care situation, adjusting to it, and adapting to the hospital environment (Oechsle et al., 2019; Ullrich et al., 2021). The demanding responsibilities of caring for seriously ill patients can adversely impact family members’ health and well-being, leading to emotional distress and burden (Choi & Seo, 2019; Huynh et al., 2023). This concern is particularly significant for women, who are often the primary caregivers, provide care for longer periods, and receive less social support and acknowledgment for their caregiving efforts (Lung et al., 2022).

Supporting families and prioritizing their needs should be a core element of palliative care (Alam et al., 2020; Bloomer et al., 2022). However, supporting family members in distressing situations is a complex task, and enhancing their well-being poses considerable challenges (Walker et al., 2023; Yıldız et al., 2024; Zhu et al., 2023). The health care system is primarily designed to cater to the needs of patients, with family members’ needs often being secondary (Røen et al., 2019).

Family experiences in palliative inpatient care reveal an absence of social, psychological, pastoral, and spiritual care for both families and patients (Walker et al., 2023). In palliative hospital care, family members seek support tailored to their needs. Moreover, they value high-quality patient care and a welcoming environment that acknowledges their presence as a source of support (Soikkeli-Jalonen et al., 2022, 2023). The importance of communication between health care professionals and families (Walker et al., 2023; Zhu et al., 2023) adds another layer of complexity, along with the stress associated with decision-making and active participation in the patient’s care (Saarinen et al., 2023; Walker et al., 2023). In palliative care, family members’ informational support is not always optimal, which can increase their anxiety and depression (Bloomer et al., 2022; Chua et al., 2020; Huynh et al., 2023; Soikkeli-Jalonen et al., 2022). Family members in palliative hospital care are dependent on health care professionals for information (Chua et al., 2020). However, although they desire information, too much information too quickly could potentially shock them (Soikkeli-Jalonen et al., 2022). Nevertheless, family members should be well-informed and actively engaged in the treatment and care process (Cheng et al., 2022; Chua et al., 2020; Soikkeli-Jalonen et al., 2022). Specifically, clear, factual, and truthful information sharing concerning patient condition, treatment, and future is essential to understand the reality of the situation and prepare for the possibility of death (Walker et al., 2023).

Moreover, the well-being of the family should be emphasized, including adaptation to the illness and its consequences, as well as social functioning and relationships (Lloyd-Williams, 2018). Research has shown that psychosocial support during palliative care can improve family members’ depressive symptoms, stress levels, caregiver burden, quality of life, self-efficacy, coping skills, and awareness levels (Yıldız et al., 2024). Family members thus require consistent and adequate assistance in navigating the health care system (Zhu et al., 2023). Practices that assess and cater to the needs of families are essential for delivering high-quality end-of-life care (Bloomer et al., 2022; Soikkeli-Jalonen et al., 2022, 2023). Self-evaluation tools and instruments can be used to objectively measure the psychosocial support experienced by family members, providing valuable information to health care staff and decision-makers. However, even though the need for psychosocial support for family members is recognized and studies have reported unmet needs (Cheng et al., 2022; Chua et al., 2020; Hashemi et al., 2018; Oechsle, 2019; Preisler et al., 2019; Ullrich et al., 2021), there is little evidence on how this support is implemented in palliative care, especially in inpatient settings (Soikkeli-Jalonen et al., 2021). Furthermore, there is a lack of evidence on how family members perceive the support they receive, as evaluated with valid and reliable instruments (Cheng et al., 2022; Michels et al., 2016).

### Aim of the Study

This study aimed to evaluate the psychosocial support received by family members in palliative inpatient care. The objective was to evaluate the implementation of psychosocial support using a self-evaluation instrument—the Family Involvement Scale–Psychosocial Support in Palliative Inpatient Care (FIS-PS-InPal). The ultimate goal of the study was to improve family support and coping, identify strengths and weaknesses in the current support system, and propose improvements to enhance the support experienced by family members.

The research questions were the following:

1. How is family members’ psychosocial support implemented in palliative inpatient care, compared with their desired support?
2. Which background factors are related to the family members’ psychosocial support, and how?

## Method

### Design

A cross-sectional study was conducted from May 2023 to September 2024 in Finnish palliative care wards. The cross-sectional design allows for a snapshot of current practices, facilitating the assessment of how well the support aligns with family members’ needs and expectations at a specific point in time. The EQUATOR-guideline “STROBE for cross-sectional studies” was utilized to ensure consistency of research implementation and reporting.

### Study Setting and Sample

The study sample comprised adult family members of adult palliative care patients from specialist palliative care wards across Finland. Specialist palliative care is offered by units that specialize in palliative care, such as palliative care centers and hospices where palliative care is the primary focus, and the staff are trained for this specific purpose (Finnish Institute for Health and Welfare, 2023). Out of the 17 specialist level wards providing palliative care as their main function in Finland at the time of the research, 16 wards, with a total of approximately 250 patient beds, participated. One ward declined to participate.

All family members were considered eligible to participate if they were adults (aged 18 and above), had adult palliative care patients in a specialist palliative care ward, and had the ability to understand and answer questions in Finnish. Their language proficiency was evaluated by the recruiting nurses at the wards. In addition, to ensure that family members could gain adequate experience of support during the ward stay, the study required the patient to have been in the ward for at least 5 days during the current treatment period. However, given that patient treatment periods in the wards were relatively short, and data collection within the 5-day limitation appeared nearly impossible, the timeline was reduced to 3 days after 6 months of data collection. The patients’ oral consent was sought for family members’ participation in the study, and the patients were asked to nominate the participating family members.

### The Family Involvement Scale–Psychosocial Support in Palliative Inpatient Care

The FIS-PS-InPal is an instrument encompassing 22 items that evaluate the psychosocial support that family members perceive during palliative inpatient care. These items represent various aspects of psychosocial support, described as subscales: psycho-emotional support (seven items), informational support (nine items), and support practices (six items). The three subscales and the total score of the instrument were represented as sum variables, while the total score of psychosocial support and sum variables in each subscale were computed by tallying the item scores and dividing by the item count.

The instrument uses a five-point response scale: 1 = significantly less than desired, 2 = less than desired, 3 = as much as desired, 4 = more than desired, and 5 = significantly more than desired, with the middle option (3) indicating optimal support. It considers whether family members perceive they have received the appropriate level of support, insufficient support, or excessive support. In addition, the instrument includes questions about background variables of the family member (age, gender, level of education, and relationship with the patient) and the patient, as reported by the family member (age, illness, time from diagnosis, length of the current hospital stay, information regarding whether the patient has been cared for in the same or another ward since the beginning; Table 1). The FIS-PS-InPal is a component of a broader instrument known as the Family Involvement Scale, which was developed and validated by the same research group conducting this study. It evaluates family members’ psychosocial support and their level of participation in inpatient care.

**Table 1.** Participants’ Demographic Characteristics and Statistically Significant Factors Related to Family Members’ Psychosocial Support.

Variable	*n*	*%*	*Mean*	FIS-PS-InPal *Med (Q1–Q3)*^a^	Psycho-emotional support *Med (Q1–Q3)*^a^	Informational support *Med (Q1–Q3)*^a^	Support practices *Med (Q1–Q3)*^a^
Gender							
Female	127	74.7		2.91 (2.63–3.00)	3.00 (2.86–3.00)	2.88 (2.44–3.00)	3.00 (2.67–3.00)
Male	42	24.7		3.00 (2.81–3.01)	3.00 (3.00–3.00)	3.00 (2.67–3.00)	3.00 (2.83–3.00)
Other/prefer not to tell	1	0.6		nr	nr	nr	nr
*Male-Female*				*p=.010* ^b^	*p=.037* ^b^	*p=.004* ^b^	*p=.049* ^b^
Age			59.22 (*SD* 13.9)	*ns* ^c^	*ns* ^c^	*ns* ^c^	*ns* ^c^
<50	42	24.7		2.86 (2.63–3.00)	3.00 (2.82–3.00)	2.78 (2.44–3.00)	2.83 (2.73–3.00)
50–59	44	25.9		2.84 (2.64–3.00)	3.00 (2.71–3.00)	2.83 (2.44–3.00)	2.83 (2.67–3.00)
60–69	37	21.8		2.96 (2.73–3.00)	3.00 (3.00–3.00)	2.90 (2.39–3.00)	3.00 (3.00–3.00)
>70	47	27.6		2.96 (2.76–3.05)	3.00 (2.68–3.00)	3.00 (2.67–3.00)	3.00 (2.67–3.00)
Educational level				*ns* ^c^	*ns* ^c^	*ns* ^c^	*ns* ^c^
Primary and lower secondary education	20	11.8		3.00 (2.70–3.03)	3.00 (2.75–3.00)	3.00 (2.57–3.00)	3.00 (2.83–3.00)
Upper secondary education	67	36.9		2.96 (2.73–3.00)	3.00 (2.86–3.00)	2.89 (2.44–3.00)	3.00 (2.83–3.00)
Bachelor’s degree	42	24.9		2.86 (2.58–2.97)	3.00 (2.71–3.00)	2.72 (2.39–3.00)	2.93 (2.67–3.00)
Master’s degree	36	21.3		2.91 (2.66–3.00)	3.00 (2.86–3.00)	2.89 (2.44–3.00)	2.83 (2.67–3.00)
Licentiate/Doctoral degree	4	2.4		2.96 (2.74–3.17)	3.00 (2.89–3.00)	2.83 (2.78–2.97)	3.00 (2.50–3.75)
Patient’s age			75.5 (*SD* 10.6)	*ns* ^c^	*ns* ^c^	*ns* ^c^	*ns* ^c^
>65	24	14.1		2.93 (2.40–3.00)	3.00 (2.46–3.00)	2.72 (2.44–3.00)	3.00 (2.50–3.13)
65–74	51	30.0		2.96 (2.76–3.00)	3.00 (3.00–3.00)	3.00 (2.56–3.00)	3.00 (2.38–3.00)
75–79	42	24.7		2.84 (2.63–3.00)	3.00 (2.86–3.00)	2.83 (2.44–3.00)	2.83 (2.67–3.00)
80–85	21	12.4		3.00 (2.81–3.09)	3.00 (2.71–3.00)	2.89 (2.78–3.00)	3.00 (2.83–3.00)
>85	32	18.8		2.93 (2.66–3.00)	3.00 (3.00–3.00)	2.78 (2.44–3.00)	3.00 (2.82–3.00)
Patient’s illness				*ns* ^c^	*ns* ^c^	*ns* ^c^	*ns* ^c^
Cancer	133	82.6		2.96 (2.66–3.00)	3.00 (2.86–3.00)	2.89 (2.44–3.00)	3.00 (2.87–3.00)
Heart disease	8	4.9		2.71 (2.57–3.00)	2.93 (2.59–3.12)	2.67 (2.36–2.86)	3.00 (2.70–3.00)
Lung disease	3	1.9		3.00 (2.91–3.01)	3.00 (3.00–3.28)	3.00 (2.78–3.00)	3.00 (3.00–3.00)
Neurological disease	8	4.9		2.93 (2.77–3.00)	3.00 (2.75–3.00)	2.89 (2.78–3.00)	3.00 (2.58–3.00)
Other	10	6.2		2.96 (2.64–3.23)	3.00 (3.00–3.00)	2.89 (2.44–3.11)	3.00 (2.67–3.83)
Time from the patient’s diagnosis *n*=115			3.0 (*SD* 3.5)	*ns* ^c^	*ns* ^c^	*ns* ^c^	*ns* ^c^
<2 years	62	53.9		2.96 (2.64–3.00)	3.00 (2.86–3.00)	3.00 (2.56–3.00)	3.00 (2.67–3.00)
2–5 years	35	30.4		2.86 (2.64–3.00)	3.00 (2.71–3.00)	2.78 (2.44–3.00)	3.00 (2.75–3.00)
6–10 years	13	11.3		2.86 (2.70–3.05)	2.86 (2.71–3.00)	2.89 (2.44–3.00)	3.00 (2.80–3.17)
>10 years	5	4.4		2.98 (2.77–3.35)	3.00 (3.00–3.11)	2.94 (2.50–3.53)	3.00 (2.88–3.50)
The length of the current hospital stay			20.1 (*SD* 24.9)	*ns* ^c^	*ns* ^c^	*ns* ^c^	*ns* ^c^
<7 days	34	22.1		2.91 (2.65–3.00)	3.00 (2.71–3.00)	2.89 (2.56–3.00)	3.00 (2.67–3.00)
7–14 days	57	37.0		3.00 (2.71–3.00)	3.00 (2.86–3.00)	3.00 (2.56–3.00)	3.00 (2.83–3.00)
15–30 days	32	20.8		2.86 (2.74–2.96)	3.00 (2.89–3.00)	2.83 (2.47–3.00)	2.83 (2.77–3.00)
>30 days	31	20.1		2.81 (2.55–3.00)	3.00 (2.71–3.00)	2.67 (2.22–3.00)	3.00 (2.33–3.00)
Patient has been cared at the same ward before				*ns* ^b^	*ns* ^b^	*ns* ^b^	*ns* ^b^
Yes	44	28.0		2.96 (2.77–3.05)	3.00 (2.86–3.00)	2.89 (2.56–3.00)	3.00 (2.83–3.00)
No	117	72.0		2.93 (2.63–3.00)	3.00 (2.86–3.00)	2.89 (2.44–3.00)	3.00 (2.67–3.00)
Patient has been cared at another ward before				ns^b^	ns^b^	ns^b^	ns^b^
Yes	139	86.9		2.91 (2.68–3.00)	3.00 (2.86–3.00)	2.89 (2.44–3.00)	3.00 (2.75–3.00)
No	20	12.5		2.96 (2.60–3.03)	3.00 (2.71–3.12)	2.89 (2.36–3.00)	3.00 (2.71–3.00)
Family members relation to the patient *n*=170				*p = .011* ^c^	*ns* ^c^	*p=.002* ^c^	*ns* ^c^
Child	83	48.8		2.86 (2.66–3.00)	3.00 (2.86–3.00)	2.78 (2.44–3.00)	2.83 (2.75–3.00)
Spouse/life partner	61	35.8		3.00 (2.77–3.07)	3.00 (2.93–3.07)	3.00 (2.67–3.00)	3.00 (2.83–3.00)
Parent	4	2.4		2.30 (1.96–2.88)	2.79 (1.93–3.00)	2.11 (1.47–2.75)	2.50 (2.21–2.92)
Sibling	10	5.9		2.96 (2.65–3.00)	3.00 (2.54–3.00)	2.89 (2.44–3.00)	3.00 (2.88–3.00)
Other	12	7.1		2.91 (2.48–3.03)	3.00 (2.50–3.00)	2.83 (2.56–3.00)	3.00 (2.71–3.13)
*Spouse/life partner–Child*				*p=.029* ^c^	*ns* ^c^	*p=.006* ^c^	*ns* ^c^
*Spouse/life partner–Parent*				*ns* ^c^	*ns* ^c^	*p=.045* ^c^	*ns* ^c^

*Note.* ns = not significant; nr = not reported; *SD* = standard deviation; Med = Median.

a Q1 = quartile 25%–Q3 = quartile 75%. ^b^ Mann–Whitney *U* test. ^c^ Kruskal–Wallis test with Bonferroni correction.

The instrument was developed using an applied meta-ethnographic synthesis of the following data sources: a previously established instrument for family support in cancer care that has demonstrated validity and reliability (Eriksson & Lauri, 2000), a comprehensive literature review (Soikkeli-Jalonen et al., 2021), two empirical qualitative descriptive studies focusing on the experiences of family members and health care professionals in palliative care units (Soikkeli-Jalonen et al., 2022, 2023), and relevant laws and recommendations (Act on the Status and Rights of Patients 785, 1992; Health Care Act, 2010) and guidelines from the World Medical Association (WMA). National Consensus Project for Quality Palliative Care (2018). The content was initially validated by three separate expert panels and piloted in three palliative care units, with the validity and usability of the content being deemed satisfactory.

The reliability of the instrument was evaluated with the data collected in this study, and the internal consistency of the FIS-PS-InPal was found to be excellent. The Cronbach’s alpha for the FIS-PS-InPal was .95, and that of different subscales ranged from .86 to .93, supporting the reliability of the instrument. The instrument’s development and piloting, as well as the evaluation of its psychometric properties have been reported in distinct articles.

### Data Collection

The data collection in this study was part of a larger research project using the Family Involvement Scale, which includes two separate scales to evaluate the psychosocial support and participation of the family member. The data collection was performed by a research team of four members who were not employed with the participating units. Therefore, a common data collection protocol was used among the research team members to ensure consistency and reliability of data. In addition, the research group contacted the coordinating contacts of each participating department monthly to ensure data collection progress.

Each participating ward was asked to designate two coordinating contact persons who were familiar with the patient and family’s situation. These contact persons were responsible for recruiting family members who met the inclusion criteria and keeping records of those who received the study form or declined to participate. For ethical reasons, the patients’ verbal consent was requested, and the patient was asked to nominate one or more family members to participate in the study before the relative was contacted. Subsequently, the family members’ willingness and consent to participate were sought. The contact persons provided these family members with research information, consent forms, and the questionnaire.

Recruited family members were asked to complete the FIS-PS-InPal questionnaire to evaluate the level of psychosocial support received during the patient’s current treatment period at the palliative care specialist ward. Paper questionnaires or Microsoft Forms e-questionnaires were used depending on the participant’s preference, and the former were returned in sealed envelopes. Family members and patients’ background factors were also collected through the same questionnaire (Table 1). In addition, the participating family members gathered information about the duration of the patients’ illness and the duration of the patients’ current stay at the ward.

Data collection began in May 2023, starting with the first ward and proceeding in the order in which research permissions were received from each organization. The data collection period per ward was either 1 year (*n* = 11) or until all the forms delivered to the ward had been distributed (*n* = 2) and the expected number of family members were recruited. However, some of the wards (*n* = 3) chose to opt out of data collection after 6 months due to an overwhelming data collection experience.

The participating wards were asked to report the number of patients and family members recruited, as well as those who declined to participate. Although the number of questionnaires distributed by the wards was reported, only half of the wards provided complete information about the participants’ consent or refusal. Based on this information, the units recruited about 300 family members, resulting in a response rate of 57%. Among the units that reported declines, 16 patients and five family members had declined participation.

### Data Analysis

A statistical analysis was performed using the IBM SPSS software (version 28.0.1.0). Initially, descriptive statistics was employed to characterize the data. Because the data was abnormally distributed with high skewness, they were analyzed with nonparametric tests and described using medians and quartiles. Missing values occurred randomly in over half of the variables, with one to four responses missing. As the absence of values could potentially affect the composite scores, when creating sum variables, missing values were accounted for by calculating the mean score for each respondent. In other analyses, missing values were handled by selecting the “exclude cases pairwise” option.

The support level of family members was described using medians, quartiles, and means for clarity. As extreme scores at both ends were rare, the variables measuring family members’ support were recoded into three-category variables: responses of 1 (significantly less than desired) and 2 (less than desired) were combined into one category, and responses of 4 (more than desired) and 5 (significantly more than desired) were combined into another. Therefore, a new three-category variable was created (less than desired = 1–2.499, as much as desired = 2.5–3.499, more than desired = 3.5–5). Family members’ perceived support was evaluated using this three-category variable, and the results were presented using percentages and frequencies. Differences between the sum variables were evaluated using the Related-Samples Friedman’s Two-Way Analysis of Variance by Ranks.

The correlations between family members’ perceived psychosocial support and background factors were examined using the Mann–Whitney U test for pairwise comparisons and the Kruskal–Wallis test with Bonferroni correction for multiple comparisons. The results were presented using medians, quartiles, and *p*-values, with the threshold for statistical significance set at *p* < .05.

### Ethical Considerations

Ethical approval was obtained from the Ethics Committee of Tampere University (approval number 85/2022) and the Ethics Committee of Pirkanmaa Hospital District (6/2023). Moreover, appropriate permissions for the research were sought from the participating organizations, and the data collection methods were designed considering the sensitivity and vulnerability of the participants. For ethical reasons related to handling information about patients and family members, participation of family members in the study was sought only after obtaining the patients’ permission. The research adhered to scientific practices, respecting the autonomy of the study participants, avoiding harm, safeguarding privacy, and ensuring data protection (World Medical Association, 2013). Moreover, the participants’ right to self-determination was respected by emphasizing the voluntary nature of participation and the option to withdraw at any time. Each participant was provided with information about the study and an informed consent form, and it was emphasized to the participants that their involvement would not affect any current or future care and treatment that they or the patient receive. Furthermore, the participants’ privacy and data protection were upheld when handling and storing the material (Finnish National Board on Research Integrity TENK, 2023; General Data Protection Regulation, 2016). All data collected during the study will remain confidential. To protect anonymity, the results of one individual in the dataset who had indicated their gender as “other” were not specified or reported separately.

## Results

### Characteristics of the Sample

A total of 171 family members of 162 patients from 16 specialized palliative care units participated in the study. Overall, 147 paper responses and 24 electronic responses were received. Most of the family members were women, with approximately half below 60 years old. Moreover, 48.8% of the participants were patients’ children, and approximately one-third were the patients’ spouses or partners. In addition, 85.5% had at least a secondary education, and nearly half had a bachelor’s degree or higher (Table 1). The patients’ average age was 75.5 years, and the dominant reason for palliative care was cancer (82.6%). Most of the patients had been ill for less than 5 years, and over half were hospitalized for less than 2 weeks during the current care period (Table 1). While most had been treated in other wards before, the majority were new to the current ward.

### Family Members’ Psychosocial Support Evaluated With the FIS-PS-InPal

The total support evaluated by family members using the FIS-PS-InPal was close to the optimal value (3 = as much as desired), with a median score of 2.96 (quartiles 2.68–3.00; Table 2). Family members reported the best support in the subscale of psycho-emotional support, with a median of 3.00 (quartiles 2.86–3.00) and 84.8% of responses indicating “as much as desired.” The subscale of informational support had the lowest scores, with a median of 2.89 (quartiles 2.44–3.00) and 69.6% of responses indicating “as much as desired” (Table 2). The differences between subscales were also statistically significant, as the mean score for informational support was significantly different from the other subscales (*p* = .000) and the total score (*p* = .010). Similarly, the psycho-emotional support subscale was significantly different from the total score (*p* = .005) and the informational support subscale (*p* = .000; Table 2).

**Table 2.** Family Members’ Psychosocial Support in Specialized Palliative Inpatient Care.

Subscale	*n*	Median (Q1–Q3)^a^	*M* (*SD*)	Frequency of answers		
				Less than desired^b^	As much as desired^c^	More than desired^d^
				% (*n*)	% (*n*)	% (*n*)
**FIS-PS-InPal**-total	171	2.96 (2.68–3.00)	2.82 (0.44)	14.0 (24)	81.3 (139)	4.7 (8)
Psycho-emotional support	171	3.00 (2.86–3.00)	2.88 (0.44)	11.1 (19)	84.8 (145)	4.1 (7)
Informational support	171	2.89 (2.44–3.00)	2.74 (0.51)	26.3 (45)	69.6 (119)	4.1 (7)
Support Practices	171	3.00 (2.75–3.00)	2.89 (0.50)	13.5 (23)	78.4 (134)	8.2 (14)
**Differences between groups**				*p* values with Related-Samples Friedman’s Two-Way Analysis of Variance by Ranks		
***FIS-PS-InPal-*** *total- Psycho-emotional support*				*p=.005*		
*FIS-In-Pal-total- Informational support*				*p=.010*		
*Informational support- Psycho-emotional support*				*p=.000*		
*Informational support-Support Practices*				*p=.000*		

a Q1 = quartile 25%–Q3 = quartile 75%. ^b^ Less than desired = 1–2.499. ^c^ As much as desired = 2.5–3.499. ^d^ More than desired = 3.5–5.

**Figure 1.**
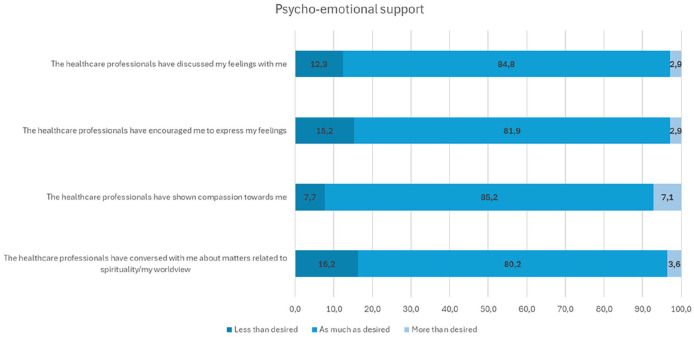
Family Members Closest to Optimal and Less-Optimally Implemented Areas of Psycho-Emotional Support.

Family members were satisfied with the psycho-emotional support they received, particularly appreciating the health care professionals’ compassion and attentive listening (Table 2). However, the least optimally realized aspect of psycho-emotional support was the occurrence of discussions without family members’ request. In addition, family members reported that they would have liked health care professionals to further encourage them to express their feelings and discuss matters related to spirituality or their worldview (Figure 1).

**Figure 2.**
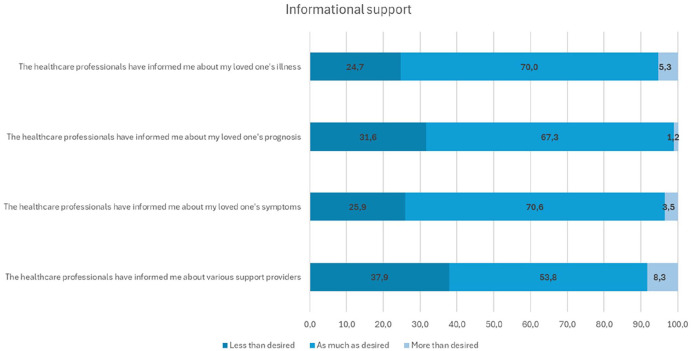
Family Members Less-Optimally Implemented Areas of Informational Support.

Informational support was the least optimally realized area of psychosocial support, with less than 70% of participants reporting that the overall informational support met their expectations (Table 2). For instance, 37.9% felt underinformed about the various support providers available, 31.6% desired more details about their loved one’s prognosis, and 29.8% wanted better insights into the practices of the department treating their loved one (Figure 2).

**Figure 3.**
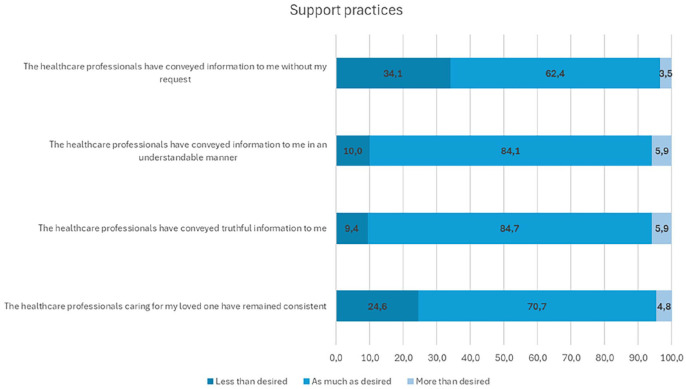
Family Members Closest to Optimal and Less-Optimally Implemented Areas of Support Practices.

Overall, family members reported that the implementation of support practices was nearly optimal (Table 2). However, despite this positive feedback, the lowest-rated aspect of psychosocial support was the provision of unsolicited information, with only 62.4% of respondents indicating they received information “as much as I desired” (Figure 3). In addition, 24.6% of family members felt that the consistency of health care professionals was less than they desired (Figure 3).

Statistically significant factors related to family members’ psychosocial support included the family members’ gender and their relation to the patient (Table 1). Men reported scores closer to optimal support (3 = as much as desired) in every area of measured psychosocial support (FIS-PS-InPal total, *p* = .010; psycho-emotional support, *p* = .037; informational support, *p* = .004; and support practices, *p* = .049), while the median scores for women were lower (Table 1). In addition, spouses/partners reported scores statistically significantly closer to optimal compared with children (FIS-PS-InPal total [*p* = .045] and informational support [*p* = .006]) and parents (FIS-PS-InPal total [*p* = .029]), whose scores were lower (Table 1). There was no statistically significant difference with other background variables and psychosocial support of family members (Table 1).

## Discussion

In this study, we evaluated family members’ psychosocial support in relation to their desire during specialist palliative inpatient care using the FIS-PS-InPal instrument. The study’s results showed three main findings. First, the average support evaluated by family members using the FIS-PS-InPal was close to the optimal value, indicating that family members are adequately supported. Second, among all support areas, informational support least met the family members’ needs. Third, gender and relationship to the patient were the only background variables related to family members’ psychosocial support, with males and spouses/life partners experiencing better support than other groups.

The findings suggest that health care professionals in specialist palliative care are generally successful in providing psychosocial support, particularly psycho-emotional support. However, proactive communication requires improvement. Over one third of the family members felt that health care professionals did not initiate discussions, although they desired this proactive approach. Moreover, family members often perceive the inpatient unit as an unfamiliar environment, which creates challenges in asking for the information and support they need (Ullrich et al., 2021). Therefore, they hope for initiative and active engagement from health care professionals in offering support and opportunities for discussion, as previous research by Soikkeli-Jalonen et al. (2022) has found. Moreover, active engagement by health care professionals is essential for family members (Cheng et al., 2022; Zhu et al., 2023). Encouraging health care professionals to initiate discussions and more actively address family members’ emotional and spiritual needs could enhance overall support, as they form an important aspect of comprehensive support (Hennessy et al., 2020; Vizzotto et al., 2013).

Informational support was identified as the weakest area of psychosocial support, with some individuals being dissatisfied with how information was communicated. However, compared with earlier studies (Bloomer et al., 2022; Chua et al., 2020; Huynh et al., 2023; Soikkeli-Jalonen et al., 2022), informational support in Finnish specialist palliative care inpatient units was found to be more efficient. Successful informational support is crucial, as family members in palliative hospital care rely heavily on health care professionals for information concerning the patient’s condition, treatment options, and care (Bloomer et al., 2022; Walker et al., 2023; Zhu et al., 2023). This support enables family members to understand the progression of the illness and to participate in patient care and decision-making (Chua et al., 2020).

Family members reported that they had less information about support providers outside the inpatient units and the practices of the care unit than they desired. This lack of information may create a feeling of being an outsider in the inpatient ward. Family members often find the hospital environment unfamiliar (Ullrich et al., 2021) and may not know how to be present and participate (Saarinen et al., 2023). Health care professionals should thus initiate conversations and highlight potential support opportunities for family members to improve their comprehensive support (Bloomer et al., 2022; Walker et al., 2023). They should recognize that discussing support possibilities and broaching the topic should be part of the psychosocial support for family members. In addition, 25% of the family members wished that the health care professionals who cared for the patient had not changed. This finding indicates that a consistent care relationship between the family members and the personnel in palliative care is important for the psychosocial support of family members.

In addition, men reported better support in all areas compared with women. It is possible that women either feel they need more support or recognize and express their need for support better than men; alternatively, their support needs may not be as well addressed in palliative ward care as those of men. However, the number of male and female participants differed significantly, with three times more women than men participating, which may limit the male perspective. As women are more often informal caregivers (Lung et al., 2022), this may explain their greater participation. Furthermore, spouses and partners reported higher satisfaction with psychosocial support compared with the parents or children of the patients. This result may be because spouses and life partners are often closest to the patient and, therefore, are more frequently present at the inpatient unit and encounter staff more often. Consequently, their opportunities to interact with health care professionals, receive support, and obtain information are greater. These differences between groups highlight the need for continuous improvement in palliative care support systems to ensure that all family members’ needs are met (Alam et al., 2020; Oechsle, 2019). However, the limited number of participants in some groups representing different relations to the patient make these results primarily indicative.

### Recommendations for Future Research

This study evaluated the psychosocial support provided at a single point during inpatient treatment, specifically when the patient’s condition was temporarily or permanently deteriorating, necessitating hospital care. It is plausible that family members’ experiences may vary at different stages of the treatment period, and the need for support can fluctuate even within the same treatment phase (Yıldız et al., 2024; Zhu et al., 2023). Future research could examine the desired and perceived realization of support not only during the treatment period but also after the patient’s inpatient care has concluded or following the patient’s demise. This analysis would help determine whether the family members’ perceptions of the support they received remain consistent or evolve over time. The study also underscores the importance of clear, factual, and truthful communication about the patient’s condition, treatment, and future, which is essential for understanding the reality of the situation and preparing for the possibility of death (Walker et al., 2023). Standardizing communication practices and providing guidelines for health care professionals can facilitate consistent and satisfactory information sharing (Bloomer et al., 2022; Walker et al., 2023).

The FIS-PS-InPal instrument allows participants to express their desire for support in an individualized manner, enabling the evaluation of the support provided. In this study, responses indicated both excessive and insufficient psychosocial support, suggesting that support should be tailored to the individual situations of family members. Notably, the same amount of support can be excessive for some while insufficient for others. Hence, recognizing and valuing the unique circumstances of family members can enhance the overall effectiveness of palliative care support systems.

### Strengths and Limitations

This study has several limitations, particularly related to the study population and data collection and their impact on the quality of the findings.

First, the generalizability of the findings to other palliative care settings may be restricted. The study’s context was limited to Finland, and its findings may not be applicable to countries with different health care systems.

Second, the selection of participants had some limitations. Participants were recruited through coordinators from the wards, and it is possible that not all eligible participants were contacted. In addition, the recruitment of family members was dependent on patient permission, which may have reduced the number of family members willing to participate. The brief hospitalization periods also posed a challenge for recruitment; the wards reported the deteriorating condition of patients as the main reason for not recruiting family members. Patients entering the wards were in relatively poor condition, their treatment times were short, and their family members were too burdened to participate in the study. Therefore, it is possible that the most burdened family members, who could benefit the most from an evaluation of their support, were excluded.

Third, the data collection process encountered several challenges regarding the response rate. Although the collection period was long, the desired number of responses were not obtained. Data collection was conducted using both paper and electronic questionnaires, with 24 responses submitted electronically. Although the paper format was more popular, the electronic option was beneficial, although it did not significantly impact the response rate. Furthermore, despite the coordinators being tasked with maintaining records of those who declined or agreed to participate, these records were not documented properly, making it impossible to report the exact number of declined invitations and the actual response rate for the study.

Fourth, the five-point response scale of the FIS-PS-InPal instrument evaluates individual psychosocial support, with the middle response option being optimal and both lower and higher scores indicating undesirable support. However, respondents may misinterpret support levels, and some may have chosen higher scores reflecting better-than-expected support, indicating a positive experience and creating biased results. In addition, three times more women than men participated in the study, which may limit the male perspective. Moreover, the number of participants representing different relationships with the patient was also uneven. These unbalanced participant groups may have biased the interpretation of the significance of differences between groups when evaluating the relationship of background variables.

## Conclusion

Generally, family members were adequately supported in specialized palliative care wards, with average support close to the optimal value. Psycho-emotional support and support practices appeared to be effective and met the desires of family members. However, informational support was identified as the weakest area, indicating that efforts should be made to improve how information is communicated. Clear, factual, and timely information is crucial for family members to understand the patient’s condition and participate in care decisions.

The slight variability in family members’ satisfaction with psychosocial support underscores the importance of personalized care. By prioritizing individualized support and enhancing communication practices, health care providers can meet the needs of all family members more efficiently. This approach ensures that family members are well-informed, actively engaged, and adequately supported throughout the care process, thereby improving the overall effectiveness of psychosocial support.

## References

[bibr1-10748407251357063] Alam S. Hannon B. Zimmermann C. (2020). Palliative care for family caregivers. JCO, 38(9), 926–936. 10.1200/JCO.19.00018 32023152

[bibr2-10748407251357063] Areia N. P. Fonseca G. Major S. Relvas A. P. (2019). Psychological morbidity in family caregivers of people living with terminal cancer: Prevalence and predictors. Palliative & Supportive Care, 17(3), 286–293. 10.1017/S1478951518000044 29478419

[bibr3-10748407251357063] Bloomer M. J. Poon P. Runacres F. Hutchinson A. M. (2022). Facilitating family needs and support at the end of life in hospital: A descriptive study. Palliative Medicine, 36(3), 549–554. 10.1177/02692163211066431 34965777 PMC8972949

[bibr4-10748407251357063] Cheng Q. Xu B. Ng M. S. N. Zheng H. So W. K. W. (2022). Needs assessment instruments for family caregivers of cancer patients receiving palliative care: A systematic review [Article]. Supportive Care in Cancer, 30(10), 8441–8453. 10.1007/s00520-022-07122-2 35633413

[bibr5-10748407251357063] Choi S. Seo J. (2019). Analysis of caregiver burden in palliative care: An integrated review. Nursing Forum, 54(2), 280–290. https://doi.org/https://doi-org.ezproxy.utu.fi/10.1111/nuf.12328 30737798 10.1111/nuf.12328

[bibr6-10748407251357063] Chua G. P. Pang G. S. Y. Yee A. C. P. Neo P. S. H. Zhou S. Lim C. Wong Y. Y. Qu D. L. Pan F. T. Yang G. M. (2020). Supporting the patients with advanced cancer and their family caregivers: What are their palliative care needs? BMC Cancer, 20(1), Article 768. 10.1186/s12885-020-07239-9 PMC7429720 32799834

[bibr7-10748407251357063] Connor S. R. (2020). Global atlas of palliative care (2nd ed.). Worldwide Hospice Palliative Care Alliance. 10.1016/j.jpainsymman.2017.03.020 28797861

[bibr8-10748407251357063] Eriksson E. Lauri S. (2000). Informational and emotional support for cancer patients’ relatives. European Journal of Cancer Care, 9(1), 8–15. 10.1046/j.1365-2354.2000.00183.x 11051937

[bibr9-10748407251357063] Finnish Institute for Health and Welfare. (2023). Organising palliative care. https://Thl.Fi/En/Topics/Ageing/End-of-Life-Care/Organising-Palliative-Care

[bibr10-10748407251357063] Finnish National Board on Research Integrity TENK. (2023). The Finnish code of conduct for research integrity and procedures for handling alleged violations of research integrity in Finland. Publications of the Finnish National Board on Research Integrity TENK 4/2023.

[bibr11-10748407251357063] General Data Protection Regulation Pub. L. No. 2016/679. (2016). Regulation (EU) 2016/679 of the European parliament and of the council. https://eur-lex.europa.eu/eli/reg/2016/679/oj

[bibr12-10748407251357063] Hashemi M. Irajpour A. Taleghani F. (2018). Caregivers needing care: The unmet needs of the family caregivers of end-of-life cancer patients. Supportive Care in Cancer: Official Journal of the Multinational Association of Supportive Care in Cancer, 26(3), 759–766. 10.1007/s00520-017-3886-2[doi] 28952034

[bibr13-10748407251357063] Hennessy N. Neenan K. Brady V. Sullivan M. Eustace-Cooke J. Timmins F. (2020). End of life in acute hospital setting-A systematic review of families’ experience of spiritual care. Journal of Clinical Nursing, 29(7–8), 1041–1052. 10.1111/jocn.15164 31891203

[bibr14-10748407251357063] Huynh T. N. T. Hartel G. Janda M. Wyld D. Merrett N. Gooden H. Neale R. E. Beesley V. L. (2023). The unmet needs of pancreatic cancer carers are associated with anxiety and depression in patients and carers. Cancers, 15(22), 5307. 10.3390/cancers15225307 PMC10670364 38001567

[bibr15-10748407251357063] Lloyd-Williams M. (2018). Psychosocial issues in palliative care. Oxford University Press. 10.1093/oso/9780198806677.001.0001

[bibr16-10748407251357063] Lung E. Y. L. Wan A. Ankita A. Baxter S. Benedet L. Li Z. Mirhosseini M. Mirza R. M. Thorpe K. Vadeboncoeur C. Klinger C. A. (2022). Informal caregiving for people with life-limiting illness: Exploring the knowledge gaps. Journal of Palliative Care, 37(2). 10.1177/0825859720984564 PMC9109592 33467993

[bibr17-10748407251357063] Michels C. T. Boulton M. Adams A. Wee B. Peters M. (2016). Psychometric properties of carer-reported outcome measures in palliative care: A systematic review. Palliative Medicine, 30(1), 23–44. 10.1177/0269216315601930 26407683 PMC4708617

[bibr18-10748407251357063] National Cancer Institute. (2024). Psychosocial support. NCI Dictionary of Cancer Terms. https://www.cancer.gov/publications/dictionaries/cancer-terms/def/psychosocial-support

[bibr19-10748407251357063] Oechsle K. (2019). Current advances in palliative & hospice care: Problems and needs of relatives and family caregivers during palliative and hospice care—An overview of current literature. Medical Sciences, 7(3), 43. 10.3390/medsci7030043 30871105 PMC6473856

[bibr20-10748407251357063] Oechsle K. Ullrich A. Marx G. Benze G. Heine J. Dickel L.-M. Zhang Y. Wowretzko F. Wendt K. N. Nauck F. Bokemeyer C. Bergelt C. (2019). Psychological burden in family caregivers of patients with advanced cancer at initiation of specialist inpatient palliative care. BMC Palliative Care, 18(1), Article 102. 10.1186/s12904-019-0469-7 PMC6862724 31739802

[bibr21-10748407251357063] Preisler M. Rohrmoser A. Goerling U. Kendel F. Bär K. Riemer M. Heuse S. Letsch A. (2019). Early palliative care for those who care: A qualitative exploration of cancer caregivers’ information needs during hospital stays. European Journal of Cancer Care, 28(2), e12990–n/a. 10.1111/ecc.12990 30623515

[bibr22-10748407251357063] Røen I. Stifoss-Hanssen H. Grande G. Kaasa S. Sand K. Knudsen A. K. (2019). Supporting carers: Health care professionals in need of system improvements and education—a qualitative study. BMC Palliative Care, 18, Article 58. 10.1186/s12904-019-0444-3 PMC6636145 31311536

[bibr23-10748407251357063] Saarinen J. Mishina K. Soikkeli-Jalonen A. Haavisto E. (2023). Family members’ participation in palliative inpatient care: An integrative review. Scandinavian Journal of Caring Sciences, 37, 897–908. 10.1111/scs.13062 34958141

[bibr24-10748407251357063] Soikkeli-Jalonen A. Mishina K. Virtanen H. Charalambous A. Haavisto E. (2021). Supportive interventions for family members of very seriously ill patients in inpatient care: A systematic review. Journal of Clinical Nursing, 30, 2179–2201. https://doi.org/https://doi.org/10.1111/jocn.15725 33616267 10.1111/jocn.15725

[bibr25-10748407251357063] Soikkeli-Jalonen A. Mishina K. Virtanen H. Charalambous A. Haavisto E. (2022). Family members’ experiences of psychosocial support in palliative care inpatient units: A descriptive qualitative study [Article]. European Journal of Oncology Nursing, 61, 102201. 36240679 10.1016/j.ejon.2022.102201

[bibr26-10748407251357063] Soikkeli-Jalonen A. Mishina K. Virtanen H. Charalambous A. Haavisto E. (2023). Healthcare professionals’ perceptions of psychosocial support for family members in palliative care inpatient units: A qualitative descriptive study [Article]. Nursing Open, 10, 3018–3027. 36539588 10.1002/nop2.1548 PMC10077415

[bibr27-10748407251357063] Ullrich A. Marx G. Bergelt C. Benze G. Zhang Y. Wowretzko F. Heine J. Dickel L.-M. Nauck F. Bokemeyer C. Oechsle K. (2021). Supportive care needs and service use during palliative care in family caregivers of patients with advanced cancer: A prospective longitudinal study. Supportive Care in Cancer, 29(3), 1303–1315. 10.1007/s00520-020-05565-z 32632761 PMC7843549

[bibr28-10748407251357063] Vizzotto A. D. B. de Oliveira A. M. Elkis H. Cordeiro Q. Buchain P. C. (2013). Psychosocial Characteristics. In: Gellman M. D. Turner J. R. (Eds.), Encyclopedia of behavioral medicine. Springer. 10.1007/978-1-4419-1005-9_918

[bibr29-10748407251357063] Walker W. Efstathiou N. Jones J. Collins P. Jennens H. (2023). Family experiences of in-hospital end-of-life care for adults: A systematic review of qualitative evidence. Journal of Clinical Nursing, 32(9–10), 2252–2269. 10.1111/jocn.16268 35332593

[bibr30-10748407251357063] Wang T. Molassiotis A. Chung B. P. M. Tan J.-Y. (2018). Unmet care needs of advanced cancer patients and their informal caregivers: A systematic review. BMC Palliative Care, 17(1), 29–96. 10.1186/s12904-018-0346-9 30037346 PMC6057056

[bibr31-10748407251357063] WMA. National Consensus Project for Quality Palliative Care. (2018). Clinical practice guidelines for quality palliative care (4th ed.). National Coalition for Hospice and Palliative Care.

[bibr32-10748407251357063] World Health Organization. (2022). WHO definition of palliative care. https://www.Who.Int/Cancer/Palliative/Definition/En/

[bibr33-10748407251357063] World Medical Association. (2013). Declaration of Helsinki: Ethical principles for medical research involving human subjects. JAMA, 310(20), 2191–2194. 10.1001/jama.2013.281053 24141714

[bibr34-10748407251357063] Yıldız M. Terzioğlu C. Ayhan F. (2024). Psychosocial interventions aimed at family members caring for patients with cancer in the palliative period: A systematic review. International Journal of Nursing Knowledge, 35, 136–151. 10.1111/2047-3095.12423 36999895

[bibr35-10748407251357063] Zhu Y. Pei X. Chen X. Li T. (2023). Family caregivers’ experiences of caring for advanced cancer patients: A qualitative systematic review and meta-synthesis. Cancer Nursing, 46(4), 270–283. 10.1097/NCC.0000000000001104 35482525

